# Factors Affecting Transfer of Pyrethroid Residues from Herbal Teas to Infusion and Influence of Physicochemical Properties of Pesticides

**DOI:** 10.3390/ijerph14101157

**Published:** 2017-09-30

**Authors:** Jin-Jing Xiao, Yang Li, Qing-Kui Fang, Yan-Hong Shi, Min Liao, Xiang-Wei Wu, Ri-Mao Hua, Hai-Qun Cao

**Affiliations:** 1 School of Plant Protection, Anhui Agricultural University, Hefei 230036, China; xiaojj187012@163.com (J.-J.X.); qkfang@163.com (Q.-K.F.); liaomin3119@126.com (M.L.); 2 Provincial Key Laboratory for Agri-Food Safety, Anhui Agricultural University, Hefei 230036, China; 15155512283@139.com (Y.L.); shiyh313@163.com (Y.-H.S.); xiangweiwu@126.com (X.-W.W.); rimaohua@126.com (R.-M.H.); 3 School of Resource & Environment, Anhui Agricultural University, Hefei 230036, China

**Keywords:** infusion, transfer, herbal tea, pyrethroids, risk assessment

## Abstract

The transfer of pesticide residues from herbal teas to their infusion is a subject of particular interest. In this study, a multi-residue analytical method for the determination of pyrethroids (fenpropathrin, beta-cypermethrin, lambda-cyhalothrin, and fenvalerate) in honeysuckle, chrysanthemum, wolfberry, and licorice and their infusion samples was validated. The transfer of pyrethroid residues from tea to infusion was investigated at different water temperatures, tea/water ratios, and infusion intervals/times. The results show that low amounts (0–6.70%) of pyrethroids were transferred under the different tea brewing conditions examined, indicating that the infusion process reduced the pyrethroid content in the extracted liquid by over 90%. Similar results were obtained for the different tea varieties, and pesticides with high water solubility and low octanol–water partition coefficients (log K_ow_) exhibited high transfer rates. Moreover, the estimated values of the exposure risk to the pyrethroids were in the range of 0.0022–0.33, indicating that the daily intake of the four pyrethroid residues from herbal tea can be regarded as safe. The present results can support the identification of suitable tea brewing conditions for significantly reducing the pesticide residue levels in the infusion.

## 1. Introduction

Herbal tea, usually a good source of phytochemicals with health-promoting properties, is consumed not only as an herbal medicine, but also as a ready-to-drink beverage [1,2]. The rising consumer awareness of the role of a healthy diet in the general well-being of an individual has been the main driver of choice for most herbal teas [3]. To meet the increasing demands for these products, recent years have seen a rapid expansion in areas planted with herbal crops, which has led to increasing numbers of serious pests and diseases.

To minimize plant losses, chemical control of pests and plant diseases has been introduced worldwide, but the continued widespread overuse of pesticides has created serious acute health problems, along with local and global environmental issues, especially in the developing countries [4,5]. For example, Greenpeace published a report “Chinese Herbs: Elixir of Health or Pesticide Cocktail?—Investigation Report on Chinese Herbs and Pesticides” in 2013 [6], showing that pesticide residues were detected in 74% of the samples examined. In particular, honeysuckle (*Lonicera japonica* Thunb*.*) samples contain the highest number (26) of pesticide residues, followed by chrysanthemum (*Chrysanthemum morifolium* Ramat.) and wolfberry (*Lycium barbarum* L.). In addition, the content of pesticide residues in some samples was tens or even hundreds of times higher than the maximum residue limit (MRL) accepted by the European Union [7]. Therefore, particular attention should be paid to the dietary exposure to pesticide residues from herbal crops.

Most food processing technologies, such as harvesting, transporting, and manufacturing [8,9], among others, are known to contribute to pesticide degradation. However, there is also evidence that pesticides can be transferred from the tea matrix to the infusion during preparation [10]. The transfer rates of several kinds of pesticides, such as pyrethroids, organophosphorus compounds, neonicotinoids, carbamates, and benzoylurea, have been reported in the range from 0 to 85% [11,12]. Whereas most studies have focused on the transfer of pesticides from green tea or black tea [13] to infusions, only limited information is available on the transfer of pesticide residues from herbal tea. The only previous studies concerning the transfer of difenoconazole and azoxystrobin from chrysanthemum, which was investigated by Xue et al. [1], and that of neonicotinoids from honeysuckle, reported in our previous study [14]. Unlike green or black tea, herbal tea does not require further processing, such as fermentation [15], and can, thus, contain higher levels of pesticide residues, with additional health risks for the consumers. However, the above studies suggested that the amount of certain pesticides in the infusion of water may be within the range considered safe for the consumers. Thus, besides determining the total concentrations, studies of the risks associated with pesticides in herbal tea should also focus on the percentages of pesticides transferred during the infusion processes.

Pyrethroids were chosen as the model pesticides in this study mainly because they are widely used and their residues are usually detected in herbal crops [16]. To the best of our knowledge, the transfer rates of these pesticides from honeysuckle, chrysanthemum, wolfberry, and licorice (*Glycyrrhiza uralensis* Fisch) tea to infusion have not been reported to date. Therefore, the present study investigated the transfer rates of pyrethroid multi-residues from tea to infusion under different conditions (e.g., water temperature, tea/water ratio (TWR), and infusion intervals/times), and analyzed the relationship between the transfer rate and the properties of these pesticides. The results of this work could allow farmers to select suitable pesticides with low transfer rates and consumers to identify appropriate tea brewing conditions for reducing pesticide exposure. Moreover, the present results will support the risk assessment of pesticide residues from herbal teas.

## 2. Materials and Methods

### 2.1. Materials

Honeysuckle, chrysanthemum, wolfberry, and licorice tea leaves were purchased from the local market as the main materials. None of the studied pyrethroids were detected in the four herbal tea samples.

Unless otherwise stated, all reagents and chemicals used in this study were purchased from Xilong Chemical Co., Ltd. (Shantou, China). High-performance liquid chromatography (HPLC)-grade *n*-hexane was obtained from Tedia Company, Inc. (Fairfield, OH, USA). Solid phase extraction (SPE) Florisil cartridges (1000 mg, 6 mL) were acquired from Agela Technologies (Beijing, China). Fenpropathrin (99.2%), beta-cypermethrin (99.2%), lambda-cyhalothrin (99.2%), and fenvalerate (99.0%) standards were purchased from Ehrenstorfer GmbH (Augsburg, Germany). Standard stock solutions of the four pyrethroids were prepared and diluted in *n*-hexane, and matrix-matched standard solutions were prepared by adding appropriate amounts of stock solution to blank (pesticide-free) extracts of herbal tea and its infusion. All solutions were stored at 4 °C until use.

### 2.2. Sample Preparation

Quintuplicate sub-samples (5.0 g each) of honeysuckle, chrysanthemum, wolfberry, and licorice samples were homogenized. Recovery tests were performed by spiking the samples at 0.01, 0.05, and 0.5 mg·kg^−1^ levels. In addition, their infusion samples were spiked with three different concentration levels (0.002, 0.02, 0.2 mg·kg^−1^) of the matrix-matched standard solutions. Then, all spiked samples were left to stand for an appropriate period of time to allow the spiking solutions to penetrate the matrix.

### 2.3. Analysis of the Four Herbal Teas and Their Infusions

An exactly-weighed 5.0 g amount of spiked tea sample was placed into a 50 mL centrifuge tube, followed by addition of 5.0 g of water and 25.0 mL of acetonitrile as the extraction solvent. After stirring the tube for 30 min, 5.0 g of sodium chloride was added to help separate acetonitrile from water, followed by centrifugation at 4000 rpm for 5 min. Finally, the supernatant was transferred to a flask, and the extraction procedure was repeated with another 25.0 mL of acetonitrile. The whole extraction solution was concentrated to near dryness by vacuum evaporation at 35 °C, and then dissolved in 4.0 mL of petroleum ether. The dissolved extract was transferred onto a SPE column, which had been conditioned with 5.0 mL of petroleum ether prior to use. The analytes were eluted with 20.0 mL of a mixture of petroleum ether and ethyl acetate (98:2, *v*/*v*) and the eluate was collected. Finally, the eluate was concentrated to dryness and reconstituted in 5.0 mL of *n*-hexane for the gas chromatography with electron capture detection (GC-ECD) analysis.

To prepare the tea infusion samples, a portion (50 mL) was placed in a 250 mL separatory funnel, and 10 g of sodium chloride was added. The infusion sample was then extracted with dichloromethane (30 mL × 3) by thoroughly shaking for 10 min. The dichloromethane solution was then collected and evaporated to dryness, and the residue was re-dissolved in 5.0 mL of *n*-hexane prior to the GC-ECD analysis.

### 2.4. Infusion Process

The honeysuckle, chrysanthemum, wolfberry, and licorice samples were fortified by spraying them with the pesticides to simulate the field trials, as described by Ozbey et al. [17]. Then, the fortified samples were subjected to the infusion process before evaporating the excess of solvent at room temperature in a fume cupboard for 15 min. Infusions were prepared with tap water under different conditions, according to the typical procedures used for brewing tea in China. The effects of the water temperature was investigated as follows: three replicates (5.0 g) of each sample were infused in 250 mL of water at 60, 70, 80, 90, or 100 °C, and the infusions were then filtered through a tea strainer with a pore size of 200 μm and cooled. The influence of the infusion time was studied by 250 mL boiling water for 10 min; then, 50 mL of the water infusion was cooled, to yield the first infusion sample. Afterwards, another 250 mL × 2 aliquots of boiling water were poured into the original flask to brew the residual tea and obtain the second and third infusions. The effect of infusion processing on the transfer of the pesticides from made tea into brew was also examined after 2, 5, 10, 20, and 30 min infusion intervals. In addition, 5.0 g of samples were infused in 150, 250, 400, and 500 mL boiling water for 10 min, respectively, to investigate the influence of the TWR. Finally, both the infusion and spent tea leaves were separately analyzed for the residues using the analytical method described above. Each experiment was performed in triplicate.

### 2.5. GC-ECD Analysis

An Agilent 7890 Network GC system (Santa Clara, CA, USA) equipped with a ^63^Ni electron capture detector and a HP-5MS capillary column (30 m × 25 μm × 2.5 μm film thickness) was used for pesticide analysis. The detector and injection temperatures were maintained at 300 and 250 °C, respectively. The GC settings were as follows: the oven temperature was held at 60 °C for 1 min, then ramped at 30 °C/min to 250 °C for 5 min, and finally ramped at 3 °C/min to 280 °C for 1 min. The carrier gas was nitrogen (99.999%), and a sample volume of 2 μL was injected in the GC system in splitless mode.

### 2.6. Statistical Analysis

All reported data are expressed as the mean ± standard error. Statistical analysis was performed using the analysis of variance (ANOVA) technique, followed by Tukey’s test [18]. All figures were drawn using the Origin Pro 9.0 software (Origin Lab Corporation, Northampton, MA, USA). Differences among means were considered statistically significant for *p* < 0.05. The transfer percentage of pesticides was calculated according to Xue et al. [1]. The exposure risk was assessed according to the following relation, recommended by the Food and Agriculture Organization (FAO):

$$\mathrm{EER}=\frac{LP\times HR}{bw\times ADI}$$

where:

(1) EER is the estimated exposure risk 

(2) *LP* is the highest large portion (kg·day^−1^ of food)

(3) *HR* is the highest residue level (mg·kg^−1^) 

(4) *bw* is the body weight (65 kg) 

(5) *ADI* is the acceptable daily intake, whose values were 30, 2, 20, and 7 mg·kg^−1^ bw^−1^, as obtained from the GB 2763-2014, Beijing, China [19].

## 3. Results and Discussion

### 3.1. Optimization of Extraction and SPE Conditions

Different extraction media, including acetonitrile, methanol, and acetone, were tested to identify the optimal solvent. The average recoveries of the pesticide residues from herbal teas, obtained with the three solvents above, were in the ranges of 77.4–82.4%, 45.3–55.8%, and 68.3–74.2%, respectively (Table S1), showing that the best results were obtained using acetonitrile. In the case of the tea infusions, dichloromethane is the most popular solvent for the recovery of pyrethroids from infusions [20,21]. In this study, the use of dichloromethane as extraction solvent resulted in less interference from other compounds in the residue analysis of the infusion sample; hence, no additional cleanup procedures were needed after the extraction with dichloromethane. 

SPE has been widely used as a cleanup technique to remove matrix co-extractives from green tea samples [22]. In this study, in order to obtain good recoveries and purification, a Florisil SPE cartridge was employed, and 5.0 mL × 5 aliquots of petroleum ether/ethyl acetate (98:2, *v*/*v*) mixture were used to elute the target pesticides and then collect each fraction. The results show that the recoveries of the first four fractions were higher than 85% (Table S2), whereas no pesticide residues were detected in the fifth fraction. Thus, 20 mL was chosen as the optimal eluent volume. The results of the recovery tests confirm the reliability of the present extraction and cleanup procedures for pesticide analysis in tea samples.

### 3.2. Method Validation

The matrix-matched calibration solutions were used for quantification, and no interfering peaks were observed in the control samples (Figure S1). Satisfactory linearity was obtained for the GC peak areas vs. calibration plots, with correlation coefficients R^2^ > 0.999, for the GC-ECD analysis of the four pesticides at concentrations ranging from 0.005 to 0.5 mg·kg^−1^ (Table 1). The accuracy of the method was verified by measuring the recovery from blank herbal tea at pesticide concentrations of 0.01, 0.05, and 0.5 mg·kg^−1^, and 0.02, 0.02, and 0.2·mg·kg^−1^ for tea infusion samples, with five replicates for each concentration. Satisfactory accuracy was obtained for all concentrations, with recoveries of the four pesticides of 80.2–110.5% from tea samples, and 80.2–115.4% from tea samples, which are within the range expected for residue analysis. Standard deviations (RSDs) < 11.23% for all pesticides demonstrate that the present method is reliable enough for the routine analysis of the pesticide residues examined in this study.

The LOD and LOQ values were defined as the pesticide concentrations for which the signal-to-noise (S/N) ratio was three and 10 times above the blank signal, respectively, at the lowest concentration levels for each matrix [23]. In this work, the LODs of the four pesticides were estimated to be 2.0 and 5.0 ng·mL^−1^ for tea and tea infusion samples, respectively, and the corresponding LOQs were 10.0 ng·mL^−1^ in both cases. The LOD and LOQ values suggest that method is also sufficiently reliable for detecting the expected concentrations of the examined residues during processing.

### 3.3. Brewing Efficiency of Pyrethroids from Tea into Infusion

Due to the well-known influence of food processing on the pesticide residue levels, various studies have taken into account the effect of food processing to estimate the dietary risks associated with tea consumption. As tea is usually steeped prior to consumption, the infusion process could reduce the content of residues absorbed by consumers by about 70%. Nevertheless, the tea brewing method plays an important role in the pesticide transfer rate. Numerous studies have indicated that the transfer rate of pesticide residues from prepared tea to infusion is affected by several factors, including water temperature, TWR, and infusion intervals/times. Therefore, we investigated the effect of these factors on the transfer of pesticide residues during the brewing process of the four herbal teas examined in this work.

**Figure 1:**
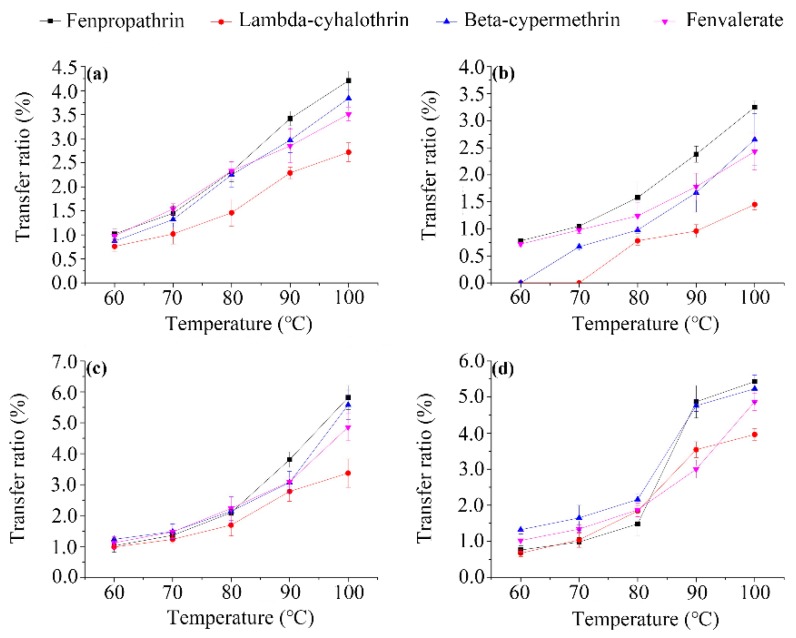
Effect of water temperature on the transfer ratio (defined as the ratio of the amounts of residues in tea and water) of the four pyrethroid residues from wolfberry (**a**); licorice (**b**); honeysuckle (**c**); and chrysanthemum (**d**) tea leaves to infusion.

**Effect of water temperature**. Several studies demonstrated that the water solubility of pesticide residues in a diluted tea brew depends on the temperature [24]. In this study, the effect of the infusion process on the transfer of the pesticides from made tea into brew was determined under various temperatures (60, 70, 80, 90, and 100 °C). As shown in Figure 1, the residual level was different for each pesticide and the transfer rate increased with increasing temperature. The increase in solubility with the water temperature is a critical factor for the transfer rate [25], which may explain the observed temperature dependence of the residue levels in the processed infusions. The increasing trends in the measured transfer rates of pesticide are in agreement with previous results reported by Cho et al. [26]. Fenpropathrin translocation to the tea infusion was the highest (0.76–5.82%), followed by beta-cypermethrin (0–5.58%) and fenvalerate (0.72–4.87%), whereas the transfer rates of lambda-cyhalothrin (0–3.96%) were comparatively lower. However, no significant differences were observed between pesticides or matrices at the same temperature, possibly because the extraction rate of the pesticides is >90% and, thus, no longer sensitive to changes in their water solubility [27]. The data also reveal that the leaching ratio varies with the type of pesticide, a trend comparable to that observed for neonicotinoids in honeysuckle [14] and difenoconazole and azoxystrobin in chrysanthemum [1]. A low leached content was measured, probably due to the influence of the water temperature on the volatilization of the four compounds. Therefore, the traditional practice of over-boiling tea leaves could lead to a higher transfer of pesticides from tea to brew, and should be discouraged. The present findings suggest an optimal water temperature of 60 °C for tea infusion.

**Figure 2:**
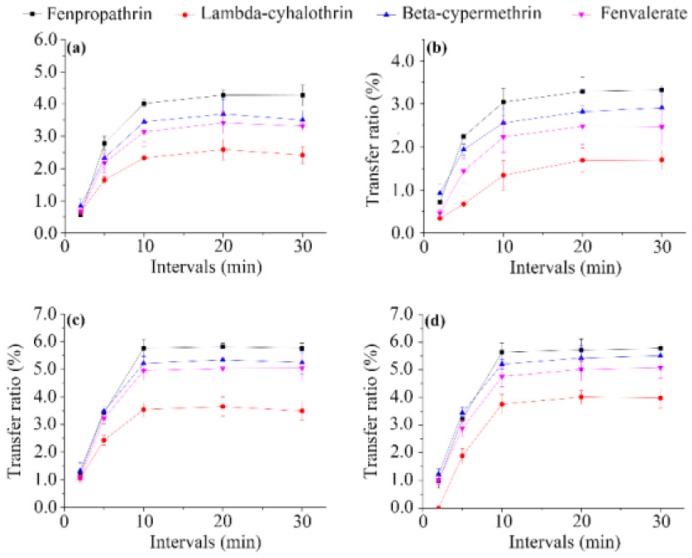
Effect of infusion interval on transfer ratios of the four pyrethroid residues from wolfberry (**a**); licorice (**b**); honeysuckle (**c**); and chrysanthemum (**d**) tea to infusion.

**Effect of infusion interval**. To investigate the effect of the infusion interval on the content of pesticide residues in made tea, different infusion intervals (2, 5, 10, 20, and 30 min) were examined (Figure 2). The proportion of pesticides released from tea leaves into brew increased gradually with increasing infusion interval, before slowing down and reaching a plateau after 10 min of infusion, accounting for 0.56–5.81% of fenpropathrin, 0.84–5.51% of beta-cypermethrin, 0.46–5.08% of fenvalerate, and 0–4.02% of lambda-cyhalothrin. Lower transfer ratios of the pesticide residues were observed for licorice tea compared with the other matrixes. A similar trend was observed in the analysis of the water temperature dependence described above, and may be due to the presence of some natural components in licorice, such as pectin, starch, and proteins, which decrease the release of contaminants from plant tissue [28]. These findings highlight the significant influence of the infusion interval on the transfer of the pesticides, and further suggest that licorice can be considered as a relatively safer herbal tea.

**Figure 3:**
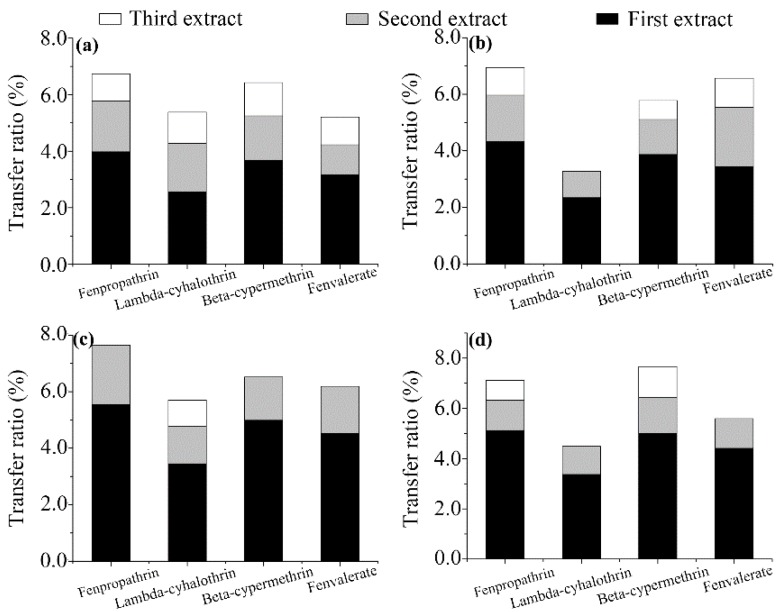
Effect of infusion time on the transfer ratios of the four pyrethroid residues from wolfberry (**a**); licorice (**b**); honeysuckle (**c**); and chrysanthemum (**d**) tea to infusion. Missing white bars denote that no corresponding residues were detected in the third infusion.

**Effect of infusion time**. The effect of the infusion time in boiled water on the release of the pyrethroids was investigated as described in the Experimental section. Going from the first to the third infusion (see Section 2.4), the transferred proportions (relative to the initial concentration) of the pesticides decreased, as shown in Figure 3. The results show that the release rates of the pyrethroids in boiled water decreased with increasing infusion time, and the highest transfer was observed during the first infusion (Figure 3). The total transfer rates measured for the second and third infusions were up to 73% lower than that of the first infusion. Fenpropathrin (6.74–7.65%) showed relatively high transfer rates from all tea varieties, except chrysanthemum, whereas lambda-cyhalothrin (3.28–5.70%) showed relatively low transfer rates. The first step of some complex tea preparation procedures involves washing tea leaves in water [29]. In this study, transfer ratios of 2.11% or less were observed in the second infusion, and lower or zero transfer ratios were found in the third infusion, indicating that the washing practice can reduce human exposure to pesticides. In summary, the infusion time was found to affect the pesticide transfer rate, and fenpropathrin and beta-cypermethrin were the most affected.

**Figure 4:**
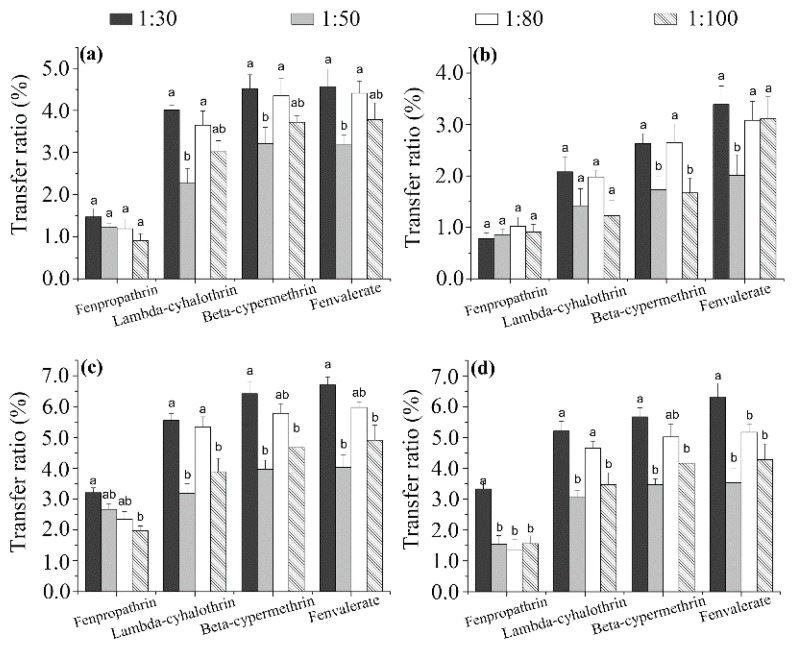
Effect of tea/water ratio on the transfer ratios of the four pyrethroid residues from wolfberry (**a**); licorice (**b**); honeysuckle (**c**); and chrysanthemum (**d**) tea to infusions. Different minor case letters (a, b) at the top of the columns mean significant differences of transfer ratio at a *p* value of 0.05.

**Effect of tea/water ratio**. Whereas most studies have focused on the effect of the factors discussed above (infusion time and intervals, water temperature, tea variety), the influence of the TWR has rarely been investigated. In this study, TWR ratios of 1:30, 1:50, 1:80, and 1:100 (*w*/*v* tea infusion) were investigated, and the results are shown in Figure 4. The transfer ratio of the pyrethroids increased with decreasing TWR, consistent with the findings of previous studies [30]. It should be noted that the concentrations of pyrethroids in the infusion decreased with decreasing TWR, which could increase the degree of dispersion of made tea in the infusion and, thus, affect the transfer ratio. The transfer ratio with TWR = 1:50 was 0.35–2.47 times higher than that with TWR = 1:30, but did not change significantly among the TWR = 1:50, 1:80, and 1:100 samples, implying that the effect of the TWR on the transfer ratio is only significant for TWRs higher than 1:50. In addition, the transfer percentage of fenpropathrin was significantly higher than that of lambda-cyhalothrin, probably due to their water solubility (Ws) and partition coefficient (log K_ow_) values (Table S3) [31]. 

### 3.4. Relationship between Transfer Rates and Physicochemical Properties of Pesticides

In traditional tea brewing methods, the transfer rate of pesticides from tea to infusion depends on their physicochemical properties, including vapor pressure, Ws, and the partition [10]. Lin et al. reported a decrease between 4.31% and 31.7% in the transfer rate of polycyclic aromatic hydrocarbons with increasing octanol–water partition coefficient [30]. In addition, continuous boiling and increased brewing times can lead to a higher transfer of pesticides into the tea infusion [12]. As reported by Ozbey and Uygun [17], the transfer rate of chlorpyrifos varies between 11% and 91% for brewing times between five and 20 min. Although the transfer rates from tea to its infusion of more than 20 pesticides have been reported, a number of pesticides with unknown transfer rates still exist. The transfer rates also depend on the tea types and brewing methods. Therefore, further investigations of the transfer rates of pesticide residues from tea to infusion are needed.

To analyze the relationship between the transfer rate and the properties of the pesticides examined in this study, the following tea brewing conditions were employed: TWR = 1:50 and 10 min infusion intervals in the first brewing. The results are shown in Table S3.

**Figure 5:**
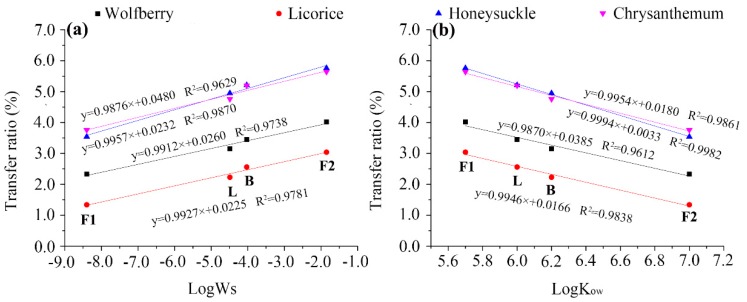
Relationship between water solubility (log Ws) (**a**) or partition coefficient (log Kow) (**b**) and the transfer rates of pesticides during tea brewing. The F1, L, B, and F2 labels in the figure represent fenpropathrin, lambda-cyhalothrin, beta-cypermethrin, and fenvalerate, respectively.

As water is the main carrier of pesticides during tea brewing, the extraction rate of pesticide residues in water has been found to be related to their water solubility. Most studies have demonstrated that the transfer rates increased as the Ws of the pesticides increases [20,32,33]. However, low transfer rates (<5%), insensitive to variations in water solubility, were observed when the Ws of the pesticides was lower than 10 mg·L^−1^ [26]. In this study, a clear linear relationship was found between the logarithm of Ws and the transfer rate of the four pesticides (Figure 5a), with correlation coefficients higher than 0.96 in all cases, indicating a good linearity.

The second main factor affecting the transfer rate of the pesticides is their octanol–water partition coefficient. Pesticides with larger K_ow_ values would strongly associate with tissues and would not move to the surrounding water, thus resulting in a minimum transfer to the infusion [34]. In this study, the transfer rates of the pesticides showed a negative correlation with log K_ow_ (Figure 5b). The R^2^ values ranged from 0.9612 to 0.9982, respectively, indicating a good linearity.

These results further confirm that the transfer rate mostly depends on the water solubility and partition coefficient parameters, and indicate that relatively high water solubilities and low partition coefficients result in high transfer rates of pyrethroids from made tea to infusion. This is also consistent with the findings discussed above.

### 3.5. Health Risk Assessment of the Pyrethroid Residues in Infusions

We assessed the health risks associated with consuming infusions of the four herbal tea varieties contaminated with the four pyrethroids. According to the pharmacopoeia of the People’s Republic of China, the recommended LPs of honeysuckle, chrysanthemum, wolfberry, and licorice are 15, 10, 12, and 10 g·day^−1^, respectively. The HRs of fenpropathrin, lambda-cyhalothrin, beta-cypermethrin, and fenvalerate in our study were 0.34, 0.027, 0.0032, and 0.010 mg·kg^−1^ for wolfberry, 0.34, 0.016, 0.0029, and 0.013 mg·kg^−1^ for licorice, 0.38, 0.029, 0.0033, and 0.012 mg·kg^−1^ for honeysuckle, and 0.36, 0.022, 0.00383, and 0.011 mg·kg^−1^ for chrysanthemum, respectively, based on a worst-case scenario assumption. 

The calculated EER for the four pyrethroids are shown in Table S4. The EER values (0.32) are much lower than 1, implying that the exposure risk to the present pesticides in infusions might be not significant. The data suggest that, although the residues can transfer from herbal tea to the tea infusion, the pesticide residue contents in the latter might be in the safe range. In particular, our calculations assume a worst-case scenario, which is unlikely to represent the real conditions. However, human exposure to pesticide residues could occur via various routes, including soil, water, air, and biomasses, among others [35], and their accumulation in the organism could have harmful effects. For this reason, an exhaustive study of the pesticide daily intake dose based on all possible exposure pathways is required in order to achieve a more accurate understanding of the associated health risks.

## 4. Conclusions

An easy, rapid, and selective GC-ECD method was established for the simultaneous determination of various pesticides, including fenpropathrin, lambda-cyhalothrin, beta-cypermethrin, and fenvalerate. The recovery tests highlighted the good accuracy and repeatability of the method, and the method was within the acceptable range for residue determinations. The mere presence of pesticide residues in tea does not necessarily entail that they would become toxic. Our analysis found that only a negligible or small percentage of pesticides were leached into the tea infusion, suggesting that the daily residue intake of the four pesticides from herbal tea would be safe. Furthermore, the extent of leaching of a pesticide mostly depends on its water solubility and partition coefficient. The pesticide transfer rates were also affected by the water temperature, tea/water ratio, and infusion intervals/times. The results indicate that discarding the water of the first brew and/or brewing tea with a water temperature of 60 °C may help in reducing the pesticide content in the infusion.

## Figures and Tables

**Table 1:** Analytical recoveries of the four pyrethroids from different herbal tea and infusion samples (*n* = 5).

Pesticide	Matrix	Fortified Level (mg·mL^−1^)	Average Recovery ± SD ^a^ (RSD ^b^)			
			Wolfberry	Licorice	Honeysuckle	Chrysanthemum
Fenpropathrin	Herb tea	0.5	96.5 ± 7.4 (5.3)	85.1 ± 6.2 (4.3)	90.3 ± 3.9 (5.4)	84.5 ± 3.7 (5.6)
		0.05	94.2 ± 3.9 (4.4)	94.5 ± 4.8 (8.1)	96.3 ± 4.1 (5.0)	93.3 ± 4.6 (7.7)
		0.01	101.6 ± 6.3 (6.7)	93.5 ± 6.0 (10.2)	98.4 ± 4.4 (7.3)	89.4 ± 4.0 (9.5)
	Tea infusion	0.2	101.9 ± 4.2 (5.0)	90.3 ± 3.9 (4.3)	85.9 ± 5.8 (4.3)	91.2 ± 9.0 (7.0)
		0.02	90.9 ± 7.1 (8.3)	92.4 ± 8.0 (6.3)	89.8 ± 7.7 (4.4)	80.9 ± 7.1 (8.5)
		0.002	112.4 ± 5.3 (3.0)	102.4 ± 4.1 (9.3)	94.5 ± 6.0 (7.2)	102.6 ± 5.0 (3.2)
Lambda-cyhalothrin	Herb tea	0.5	96.2 ± 4.2 (9.2)	110.5 ± 4.7 (6.2)	85.3 ± 9.7 (5.2)	98.3 ± 9.5 (6.6)
		0.05	84.5 ± 5.6 (8.3)	85.9 ± 7.2 (8.3)	90.2 ± 11.8 (7.5)	107.9 ± 8.3 (8.7)
		0.01	95.1 ± 8.2 (9.1)	92.8 ± 6.2 (9.1)	94.5 ± 9.7 (9.1)	102.4 ± 10.1 (6.9)
	Tea infusion	0.2	109.5 ± 8.9 (5.4)	89.5 ± 6.1 (7.3)	87.2 ± 3.6 (5.0)	90.0 ± 7.0 (5.4)
		0.02	103.8 ± 7.9 (10.5)	103.8 ± 7.3 (8.3)	98.2 ± 4.6 (6.9)	93.6 ± 9.5 (10.5)
		0.002	110.4 ± 3.9 (8.4)	102.4 ± 6.6 (9.6)	89.4 ± 5.7 (9.4)	101.4 ± 6.2 (8.34)
Beta-cypermethrin	Herb tea	0.5	89.3 ± 9.2 (5.7)	82.0 ± 8.5 (5.7)	86.3 ± 6.9 (5.3)	88.4 ± 4.5 (6.4)
		0.05	85.2 ± 3.7 (6.2)	80.2 ± 3.2 (6.3)	94.7 ± 3.9 (6.6)	90.5 ± 4.1 (1.2)
		0.01	92.7 ± 9.8 (6.2)	91.9 ± 5.5 (9.3)	89.6 ± 4.9 (6.8)	93.6 ± 6.9 (9.9)
	Tea infusion	0.2	115.4 ± 3.6 (5.6)	81.4 ± 4.7 (4.4)	87.4 ± 8.3 (5.7)	95.4 ± 9.9 (5.6)
		0.02	101.5 ± 4.1 (5.2)	91.5 ± 7.6 (6.8)	93.6 ± 6.9 (6.3)	81.5 ± 7.9 (11.0)
		0.002	88.4 ± 3.8 (5.9)	98.4 ± 5.8 (7.6)	91.2 ± 9.0 (9.3)	89.6 ±4.9 (5.9)
Fenvalerate	Herb tea	0.5	90.1 ± 4.0 (6.1)	102.8 ± 9.6 (10.1)	94.2 ± 3.9 (5.3)	89.8 ± 9.5 (6.5)
		0.05	96.2 ± 3.7 (2.3)	96.2 ± 8.4 (7.3)	102.4 ± 7.9 (8.4)	95.7 ± 9.3 (8.5)
		0.01	103.5 ± 3.5 (7.6)	84.3 ± 4.7 (5.6)	97.2 ± 3.5 (9.3)	98.3 ± 3.7 (9.1)
	Tea infusion	0.2	89.2 ± 5.8 (6.4)	80.2 ± 3.8 (9.4)	94.3 ± 4.9 (4.2)	87.3 ± 8.6 (6.4)
		0.02	88.6 ± 6.9 (5.2)	98.5 ± 7.9 (7.2)	96.2 ± 6.9 (5.5)	98.3 ± 7.8 (5.2)
		0.002	113.5 ± 7.7 (7.1)	91.4 ± 4.7 (8.4)	95.4 ± 5.8 (7.3)	103.5 ± 6.7 (7.1)

^a^ Standard deviation (%); ^b^ Relative standard deviation (%).

## Supplementary Materials

The following materials are available online at www.mdpi.com/1660-4601/14/10/1157/s1, Figure S1. Typical GC-ECD chromatograms of fenpropathrin, lambda-cyhalothrin, beta-cypermethrin, and fenvalerate in infused tea. Table S1. Recovery ratios and RSDs of pyrethroids by different extraction solvents (*n* = 3). Table S2. Dependence of recovery ratios of the four pyrethroids on the volume of elution solvent (*n* = 3). Table S3. Transfer rate of the four pyrethroids in different herbal teas and corresponding physicochemical properties. Table S4. Estimated exposure risk to the four pyrethroids from honeysuckle, chrysanthemum, wolfberry, and licorice tea.
