# Supplementary material for: Factors Affecting Transfer of Pyrethroid Residues from Herbal Teas to Infusion and Influence of Physicochemical Properties of Pesticides
Source: Int J Environ Res Public Health. 2017 Sep 30;14(10):1157. doi: 10.3390/ijerph14101157 (PMC5664658; doi:10.3390/ijerph14101157)
Supplement: Supplementary file 1 [file ijerph-14-01157-s001.pdf]

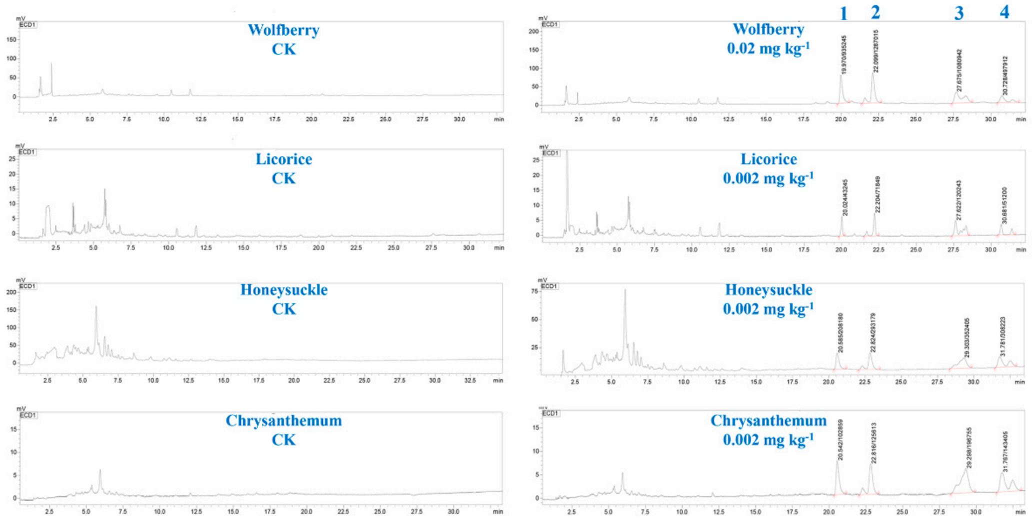


**Figure S1.** Typical GC-ECD chromatograms of fenpropathrin, lambda-cyhalothrin, beta-cypermethrin, and fenvalerate in infused tea.

**Table S1.** Recovery ratios and RSDs of pyrethroids by different extraction solvents ($n$ = 3)

| Pesticides | Acetonitrile | | Methanol | | Acetone | |
|---|---|---|---|---|---|---|
| | Average recovery ± SD [a] (%) | RSD (%) [b] | Average recovery (%) | RSD (%) | Average recovery (%) | RSD (%) |
| Fenpropathrin | 78.2 ± 4.6 | 3.24 | 45.3 ± 1.9 | 4.33 | 74.2 ± 4.0 | 6.22 |
| Lambda-cyhalothrin | 77.4 ± 5.7 | 4.23 | 51.2 ± 5.5 | 3.23 | 70.1 ± 2.1 | 2.39 |
| Beta-cypermethrin | 80.2 ± 9.3 | 5.22 | 47.6 ± 9.3 | 6.44 | 76.1 ± 2.6 | 4.38 |
| Fenvalerate | 82.4 ± 4.4 | 3.42 | 55.8 ± 3.2 | 4.97 | 68.3 ± 9.7 | 4.32 |

[a] Standard deviation; [b] Relative standard deviation for reproducibility in %.

**Table S2.** Dependence of recovery ratios of the four pyrethroids on the volume of elution solvent ($n$ = 3)

| Pesticides | Recoveries of fraction 1 (%) | Recoveries of fraction 2 (%) | Recoveries of fraction 3 (%) | Recoveries of fraction 4 (%) | Recoveries of Fraction 5 (%) | Recoveries of Sum (%) |
|---|---|---|---|---|---|---|
| Fenpropathrin | 20.3 ± 1.7 [a] | 50.8 ± 3.8 | 15.2 ± 3.6 | ND [b] | ND | 86.3 |
| Lambda-cyhalothrin | 14.5 ± 0.7 | 64.3 ± 8.4 | 9.2 ± 1.0 | 1.2 ± 0.5 | ND | 89.2 |
| Beta-cypermethrin | 27.3 ± 1.4 | 61.3 ± 4.9 | 5.4 ± 1.3 | ND | ND | 94.0 |
| Fenvalerate | 26.8 ± 2.9 | 45.2 ± 6.2 | 19.5 ± 4.5 | 2.3 ± 1.1 | ND | 93.8 |

[a] mean residual rate ± Standard deviation (SD) (%); [b] Not detected.

**Table S3.** Transfer rate of the four pyrethroids in different herbal teas and corresponding physicochemical properties

| Pesticides | transfer ratio ± SD [a] % | | | | Ws [b] (mg·$L^{-1}$) (25°C) | $LogK_{ow}$ [c] |
|---|---|---|---|---|---|---|
| | Wolfberry | Licorice | Honeysuckle | Chrysanthemum | | |
| Fenpropathrin | 4.02 ± 0.85 a [d] | 3.04 ± 0.16 a | 5.76± 0.33 b | 5.64± 0.32 b | $1.4\times10^{-2}$ | 5.7 |
| Lambda-cyhalothrin | 2.33 ± 0.26 a | 1.34 ± 0.21 a | 3.54± 0.41 b | 3.76± 0.18 b | $4.0\times10^{-9}$ | 7.0 |
| Beta-cyhalothrin | 3.45 ± 0.35 a | 2.56 ± 0.15 a | 5.21± 0.22 b | 5.20± 0.42 b | $9.3\times10^{-5}$ | 6.0 |
| Fenvalerate | 3.15 ± 0.18 a | 2.23 ± 0.10 a | 4.95± 0.40 b | 4.76± 0.13 b | $3.2\times10^{-5}$ | 6.2 |

[a] Standard deviation; [b] water solubility; [c] Octanol-water partition coefficient; [d] Different lower case letters at the top of the columns mean significant differences in the transfer ratio at a *p* value of 0.05. Ws and $LogK_{ow}$ were obtained from Tomlin, 1997.

**Table S4.** Estimated exposure risk to the four pyrethroids from honeysuckle, chrysanthemum, wolfberry, and licorice tea.

| Matrix | Pesticides | MRL [a] (mg kg$^{-1}$) | ADI [b] (mg kg$^{-1}$ bw$^{-1}$) | Transfer ratio [c] (%) | EER |
|---|---|---|---|---|---|
| Fenpropathrin | Wolfberry | 5 | 30 | 6.74 | 0.21 |
| | Licorice | | | 6.95 | 0.18 |
| | Honeysuckle | | | 7.65 | 0.29 |
| | Chrysanthemum | | | 7.11 | 0.18 |
| Lambda-cyhalothrin | Wolfberry | 0.5 | 2 | 5.38 | 0.25 |
| | Licorice | | | 3.28 | 0.13 |
| | Honeysuckle | | | 5.70 | 0.33 |
| | Chrysanthemum | | | 4.49 | 0.17 |
| Beta-cyhalothrin | Wolfberry | 0.05 | 20 | 6.43 | 0.0030 |
| | Licorice | | | 5.78 | 0.0022 |
| | Honeysuckle | | | 6.52 | 0.0038 |
| | Chrysanthemum | | | 7.66 | 0.0029 |
| Fenvalerate | Wolfberry | 0.2 | 7 | 5.21 | 0.027 |
| | Licorice | | | 6.57 | 0.029 |
| | Honeysuckle | | | 6.19 | 0.041 |
| | Chrysanthemum | | | 5.59 | 0.025 |

[a] Maximum residue limit, MRL values of lambda-cyhalothrin and beta-cyhalothrin were obtained from the EU and Japan, respectively, and others were obtained from the GB 2763-2014, China (National Food Safety Standard-maximum residue limits for pesticides in food); [b] Acceptable daily intake; [c] Obtained from the total transfer ratios of the first, second and third infusions.
